# Multi-Element Exposure in a High-Altitude Páramo Mining District and Oxidative Stress Biomarkers in Gold Miners

**DOI:** 10.3390/toxics14060534

**Published:** 2026-06-20

**Authors:** Lyda Espitia-Pérez, Luz Helena Sánchez Rodríguez, Hugo Brango, Pedro Espitia-Pérez, Dina Ricardo-Caldera, Laura Andrea Rodríguez-Villamizar, Álvaro J. Idrovo

**Affiliations:** 1 Grupo de Investigación Biomédica y Biología Molecular, Facultad de Ciencias de la Salud, Universidad del Sinú, Montería 230001, Colombia; pedrojespitia@unisinu.edu.co; 2 Grupo de Investigación en Compuestos Orgánicos de Interés Medicinal, Escuela de Microbiología, Universidad Industrial de Santander, Bucaramanga 680002, Colombia; lsanchez@uis.edu.co; 3 Departamento de Matemáticas, Facultad de Educación y Ciencias, Universidad de Sucre, Sincelejo 700003, Colombia; 4 Grupo de Investigación Enfermedades Tropicales y Resistencia Bacteriana, Facultad de Ciencias de la Salud, Universidad del Sinú, Montería 230001, Colombia; dinaricardoc@unisinu.edu.co; 5 Grupo de Investigación en Demografía, Salud Pública y Sistemas de Salud, Departamento de Salud Pública, Escuela de Medicina, Universidad Industrial de Santander, Bucaramanga 680002, Colombia; laurovi@uis.edu.co (L.A.R.-V.); idrovoaj@uis.edu.co (Á.J.I.)

**Keywords:** environmental exposure, occupational exposure, mining, altitude, metals, heavy, oxidative stress, glutathione, biomarkers

## Abstract

Artisanal and small-scale gold mining (ASGM) generates complex metal mixtures, yet their biological effects remain poorly characterized in high-altitude populations, where occupational exposure occurs against a hypoxic environmental background. This study evaluated 49 occupationally exposed gold miners from the Vetas–California mining district, near the Santurbán páramo in Colombia, and 25 non-exposed individuals from a comparable high-altitude area. Hair concentrations of essential and toxic elements were quantified by ICP-MS, and serum catalase (CAT), superoxide dismutase (SOD), reduced glutathione (GSH), oxidized glutathione (GSSG), and the GSH/GSSG ratio were assessed. Miners showed a distinct multielement profile, with a higher toxic-metal burden and a dominant mixture mainly characterized by Fe, Mn, As, Pb, Cd, and Hg. CAT and SOD activities did not differ markedly between groups, although SOD activity decreased along the main exposure gradient among exposed workers. In contrast, glutathione-related biomarkers showed a more consistent exposure-related pattern, with higher GSSG and a lower GSH/GSSG ratio, suggesting a shift toward a more oxidized glutathione redox status. Together with positive within-group associations between selected elements and the GSH/GSSG ratio, these results are consistent with a mixture-associated perturbation of glutathione redox homeostasis, with heterogeneous adaptive responses. Overall, this study supports the use of integrated biomonitoring strategies and highlights glutathione-related markers as potential indicators of early redox perturbation in high-altitude mining populations.

## 1. Introduction

Artisanal and small-scale gold mining (ASGM) is one of the most widespread informal economic activities in low- and middle-income countries and a significant source of global mercury emissions and related health risks [1,2]. Although ASGM provides income and employment for millions of people worldwide, mining operations are often conducted with rudimentary technologies and limited regulatory oversight, resulting in heterogeneous, often poorly characterized exposure profiles. These profiles are shaped by ore-processing practices, geological matrices, and local environmental conditions, and they commonly involve mixed exposure to mercury (Hg) and other toxic elements released during ore extraction, crushing, amalgamation, and burning processes [3]. Particularly among gold miners, this multi-elemental exposure occurs through both skin contact and ingestion. The primary route of exposure, however, is through inhalation of significant concentrations of vapors during the gold processing and extraction stages [4,5]. Additionally, these contaminants can also be present in soils and mine tailings from ASGM areas, where exposure mainly occurs through the dermal route [6,7,8].

Human biomonitoring studies in ASGM communities consistently demonstrate that, in addition to Hg, miners and nearby residents are exposed to complex mixtures of arsenic (As), lead (Pb), cadmium (Cd), manganese (Mn), and other metals and metalloids [9]. Investigations in mining districts across South America—including Peru [10], Bolivia [11], Brazil [12], and Colombia [13,14]—have reported elevated concentrations of these elements in biological matrices such as urine, blood, and hair, as well as in environmental media including soil, sediments, and water.

Epidemiological and toxicological studies indicate that exposure to mixtures of chemical elements in ASGM settings is associated with a broad spectrum of adverse health effects, including neurotoxicity, respiratory impairment, cardiovascular alterations, reproductive effects, and genetic damage [15,16,17,18]. Across these outcomes, oxidative stress has emerged as a key mechanistic pathway mediating metal-induced toxicity [19,20,21]. Redox-active and redox-disrupting metals commonly present in ASGM—particularly Hg, As, Pb, and Cd—can induce reactive oxygen species (ROS), impair mitochondrial function, disrupt cellular redox homeostasis, and interfere with antioxidant defense systems [22,23,24]. In gold-mining populations, metal exposure has been associated with altered activity of key enzymatic antioxidants, including superoxide dismutase (SOD), catalase (CAT), and glutathione peroxidase (GPx), as well as with increased markers of oxidative damage, supporting their use as early indicators of biological effect [25,26].

Most existing evidence on metal-related oxidative stress and its health effects in ASGM populations comes from mining settings outside high-mountain ecosystems. High-altitude gold mining introduces additional environmental and physiological stressors that may modify exposure–effect relationships. Mining activities at elevations ≥2500 m above sea level are characterized by chronic hypobaric hypoxia, compensatory erythrocytosis, increased ventilation rates, and metabolic adaptations to reduced oxygen availability [27,28]. Hypoxia itself is a recognized stimulus for increased ROS production and modulation of antioxidant enzyme activity [29,30,31], suggesting that biological responses to metal exposure in high-altitude environments may differ from those observed at lower elevations [32].

This distinction is particularly relevant in Colombia, which harbors more than half of the world’s páramo ecosystems [33] and includes one of the few high-mountain páramo regions globally where artisanal and small-scale gold mining is practiced [34,35,36]. The ecological characteristics of páramos—including fragile soils, high humidity, intense solar radiation, and limited buffering capacity—may influence metal mobility and bioavailability, thereby shaping human exposure pathways. We hypothesize that, in combination with chronic occupational exposure to metal mixtures and physiological stress associated with high altitude, these conditions may contribute to distinct biological responses and health effects among miners working in páramo environments. Consequently, occupational metal exposure in páramo ecosystems is highly relevant to human health assessment yet remains insufficiently investigated. The present study addresses this gap by quantifying internal concentrations of toxic and essential elements and evaluating their association with oxidative stress-related enzymatic markers in gold miners from the Vetas–California district, located at the boundary of the Santurbán páramo in northeastern Colombia. By integrating occupational metal exposure with oxidative stress parameters in a páramo context, this study provides context-specific evidence on early biological responses to polymetallic exposure in one of the world’s most vulnerable high-mountain ecosystems.

## 2. Materials and Methods

### 2.1. Study Design and Characteristics of the Santurbán Mining Corridor

**Figure 1:**
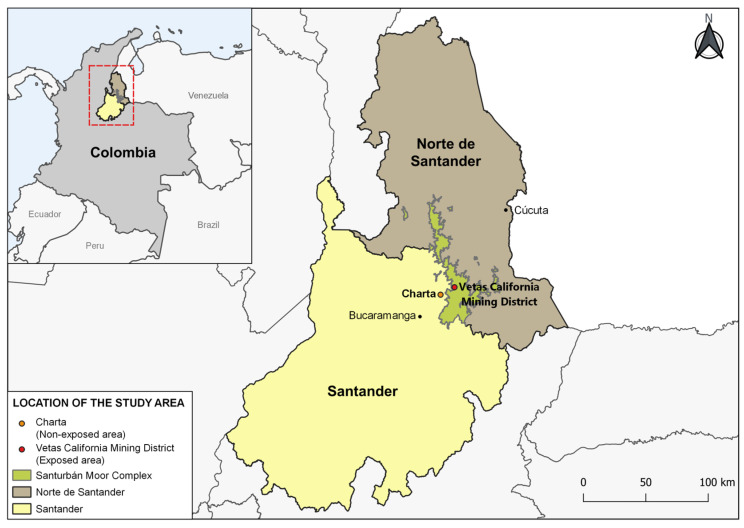
Sampling areas.

We conducted an exploratory, descriptive cross-sectional study in 2019 to characterize multi-element exposure among artisanal gold miners living in a high-altitude mining corridor in northeastern Colombia. This mining district, in the department of Santander, lies along the boundary of the Santurbán páramo, a protected high-mountain wetland ecosystem internationally recognized for its hydrologic regulation and ecological sensitivity [37]. Geological assessments indicate that Santurbán overlies one of the largest auriferous and argentiferous deposits reported for the northern Andes, with estimated resources of approximately 239.5 metric tons of gold and 2488.3 metric tons of silver, equivalent to 7.7 million and 80 million troy ounces [38], respectively. The coexistence of these mineral reserves within an ecologically fragile páramo environment has been identified as a source of recurrent socio-environmental conflict, particularly pressures from artisanal and small-scale mining on water resources and ecosystem integrity [39]. The mining district encompasses the municipalities of Vetas and California (exposed area), where gold extraction has taken place since colonial times and continues to rely predominantly on artisanal and small-scale mining practices [40]. The exposed mining area spans an approximate altitude range of 2050–3350 m a.s.l. Vetas is located at 07°18′41″ N and 72°52′20″ W at an altitude of approximately 3350 m a.s.l., with an average temperature of 9 °C, whereas California lies at 07°21′03″ N and 72°56′56″ W at 2050 m a.s.l., with an average temperature of 17 °C. Gold mining is practiced in underground deposits using tunnels that follow the vein; in this type of mining, galleries are developed as transportation routes. This type of artisanal mining causes high-level pollution of water bodies [41]. A non-exposed area was located in the same geographic region within the municipality of Charta, a non-mining municipality in the same Andean region, located at approximately 2474 m a.s.l. and 257 km from the gold mines; this area is characterized by the absence of mining or agricultural activities and has a population with similar sociodemographic characteristics (Figure 1). The non-exposed population was intentionally drawn from a similar high-altitude setting to allow the evaluation of mining-related multielement exposure within a shared physiological context.

### 2.2. Human Biomonitoring

Participants in both exposed and non-exposed areas completed a structured questionnaire that collected information on diagnosed medical conditions, including hypertension, diabetes, and cancer, as well as lifestyle characteristics, dietary habits, smoking status, frequency of alcohol consumption, the number of dental fillings, previous and current occupational exposures, duration of mining-related activities, and the use of personal protective measures. In the exposed area, eligible individuals were adults aged 18 or older, permanent residents of the Vetas–California mining district, who voluntarily agreed to participate and had worked as gold miners in the Vetas–California district since at least 2010. In the non-exposed area, eligible individuals were adults aged 18 years or older, permanent residents of Charta, with no history of gold mining or any other mining-related activities. Participants were selected from a pool of 200 individuals enrolled in a previous cross-sectional study, and frequency matching by age and socioeconomic status was used to reduce potential confounding. A total of 74 healthy adults were included in the current biomonitoring assessment: 49 occupationally exposed gold miners from the Vetas–California mining district and 25 non-exposed participants from the municipality of Charta. The final sample size reflects the demographic and logistical constraints inherent to the study context. In the Vetas–California district, artisanal gold mining is characterized by small, low-density, family-based mining groups operating within a protected high-mountain ecosystem. Because mining activities in this region occur within and around a legally protected páramo area, these operations are subject to regulatory, social, and environmental scrutiny. As a result, many miners maintain a low profile, which decreases the number of individuals who can be formally enrolled in research activities.

Similarly, the pool of eligible non-exposed participants was limited by strict inclusion criteria and sociodemographic comparability with the exposed group. These requirements significantly narrowed the pool of eligible volunteers. Rather than increasing the size of the reference group at the expense of comparability, we prioritized a carefully selected non-exposed group. Although the final sample size is modest, it is both realistic and representative of the accessible mining population in this district.

Written informed consent was obtained from all participants before sample collection. All questionnaire responses and individual metadata were systematically organized into electronic databases. No significant differences in socioeconomic status or dietary habits were observed between the exposed and non-exposed groups. After enrollment and questionnaire administration, biological samples were collected using standardized procedures to assess trace-element concentrations and oxidative stress biomarkers.

### 2.3. Blood Sample Collection and Processing

Blood samples were collected following the procedures described by Rodríguez et al. [42]. After obtaining written informed consent, peripheral blood was collected from all 74 participants. A total of 5 mL of blood was collected into serum-separation tubes containing clot activator and separator gel (Becton Dickinson (Franklin Lakes, NJ, USA), Vacutainer).

Blood samples were centrifuged at 3500 rpm for 10 min to obtain the serum fraction, which was used exclusively to determine antioxidant enzyme activities and assess circulating oxidative stress biomarkers. All serum samples were handled in accordance with standardized operating procedures, including chain-of-custody documentation, temperature monitoring during transport, and batch processing to minimize analytical variability. Internal quality assurance procedures were applied throughout sample handling and storage to ensure sample integrity and the reproducibility of oxidative stress measurements.

### 2.4. Redox Profile

For biochemical redox parameters in serum, enzymatic antioxidant activities and reduced/oxidized glutathione (GSH/GSSG) levels were measured. Serum samples were either analyzed directly or normalized to protein content using the Lowry method with bovine albumin as the standard for enzymatic activity. For glutathione measurement, serum was pre-treated with 5% sulfosalicylic acid, centrifuged (8000× *g*, 10 min) to remove cellular debris, and the supernatant was used for analysis.

Superoxide dismutase (SOD) activity was determined by the Misra and Fridovich procedure [43], with slight modifications for serum samples. The method measures adrenaline-dependent inhibition of superoxide auto-oxidation at 480 nm. Results were expressed as units of SOD activity per milligram of protein. Catalase (CAT) activity was measured in serum samples using the method described by Aebi [44]. The protocol was based on the decrease in absorbance at 240 nm due to H_2_O_2_ degradation over 1 min, as measured by a spectrophotometer. Results were expressed as units of CAT per milligram of protein.

For GSH/GSSG measurement, we used a commercially available kit from Sigma-Aldrich (St. Louis, MO, USA; Cat. No. 38185) following the manufacturer’s instructions to analyze serum samples. Results were reported as individual concentrations of GSH (mol GSH/L) and GSSG (mol GSSG/L), or as the GSH/GSSG ratio, a parameter used to assess redox status in both studied populations.

### 2.5. Hair Sample Collection, Preparation, and Processing

Hair samples were collected from participants using a previously established protocol [45]. Briefly, hair samples (250 mg) were collected from the occipital scalp region and stored in polypropylene bags, each with a previously assigned code for each participant. During laboratory processing, when sufficient material was available, the proximal hair segment was selected, and any portion in direct contact with sampling or holding materials was avoided. In underground ASGM settings, external contamination of hair may occur via deposition of mineral dust and inorganic particulate material; therefore, a detergent-based pre-washing protocol was used prior to elemental analysis. Hair samples were immersed in 2% Triton X-100, a non-ionic detergent previously used in trace-element hair analysis [46], and sonicated for 1 min to promote detachment of superficial residues. This step was followed by three sequential rinses with distilled water, 1 min each, to remove residual detergent and loosely bound external material. After drying, hair was cut using stainless-steel scissors previously cleaned with 5% HNO_3_ and distilled water before weighing. This washing sequence was selected to reduce external contamination while avoiding excessive loss of soluble endogenous elements [47,48]. About 200 mg of hair was weighed into a 15 mL polypropylene conical tube. Then, 1 mL of ultrapure concentrated nitric acid (65%) was added to the samples. Nitric-acid extraction was left at room temperature (25–28 °C) for more than 48 h with the cap not completely closed. After that, they were diluted to a final volume of 10 mL. Then, a 10% volume of a solution containing 100 µg/L of the internal standards scandium (Sc) and yttrium (Y) was added to obtain a final concentration of 90.9 ug/L of Sc and Y.

### 2.6. Analysis of Chemical Elements by Inductively Coupled Plasma Mass Spectrometry (ICP-MS)

Trace-element determinations in hair samples were performed using an Agilent 7500-A Inductively Coupled Plasma Mass Spectrometer (ICP-MS; Agilent Technologies, Santa Clara, CA, USA) with ChemStation G1834B software, version B.03.02 (U300-0009), running under Microsoft ^®^ Windows 2000 ^®^. Samples were introduced via a peristaltic pump and a concentric nebulizer under standard multielement operating conditions. Each sample underwent an initial stabilization period, followed by duplicate analytical readings to improve measurement robustness. Quantification was performed using external calibration curves prepared from certified multielement standard solutions diluted in ultrapure nitric acid. Calibration curves spanned multiple concentration points covering the expected range of trace elements in human hair and were accepted only when the correlation coefficient exceeded 0.999. Instrument drift and matrix effects were monitored and corrected using internal standards and calibration verification solutions analyzed periodically throughout each analytical run. External standard concentration ranges used for ICP-MS calibration/checking and method quantification limits (LOQ/LQ) were established for the quantified elements and are provided in Supplementary Table S3. Calibration/checking ranges are expressed as µg/L in the measured extract, whereas LOQ/LQ values are expressed in the same units as the final hair concentrations (µg/g hair). Detailed ICP-MS instrumental and analytical conditions, including sample introduction, acquisition scheme, internal standards, monitored m/z values for the retained analytes, calibration approach, and QA/QC procedures, are provided in Supplementary Table S4.

Analytical accuracy and reproducibility were ensured through a comprehensive quality assurance/quality control (QA/QC) protocol. The accuracy and precision of ICP-MS measurements were assessed using five certified/reference human hair materials, including NCS DC 73347 Human Hair (National Analysis Center for Iron and Steel–NCS Testing Technology Co., Ltd., Beijing, China), IAEA-085 and IAEA-086 Human Hair (International Atomic Energy Agency, Vienna, Austria), ERM DB001 Human Hair (European Commission Joint Research Centre, Geel, Belgium), and NIES CRM No. 13 Human Hair (National Institute for Environmental Studies, Tsukuba, Ibaraki, Japan). Certified-element recoveries were required to fall within the acceptable ranges specified for each CRM. Analytical precision was assessed by duplicate nitric-acid extraction and measurement of a subset of samples, and procedural blanks were included in each batch to estimate instrument background and method quantification limits. Internal standards were added to all samples to monitor and correct for instrumental drift. Only elements with valid CRM recoveries and within the calibrated analytical range were included in the quantitative dataset used in this manuscript.

### 2.7. Statistical Analysis

The data underwent quality control measures, such as outlier detection, consistency verification, and missing data management. Potential outliers were identified using the interquartile range (IQR) criterion, defined as observations below Q1 – 1.5 × IQR or above Q3 + 1.5 × IQR. Flagged observations were reviewed for data-entry errors, analytical inconsistencies, or implausible values. No observations were excluded because all values were considered plausible within the context of occupational and environmental metal exposure.

For trace-element measurements included in the quantitative exposure dataset, concentrations below the LOQ/LQ were assigned a value of one-half the corresponding LOQ before statistical analysis. A descriptive analysis of sociodemographic, occupational, and lifestyle characteristics was conducted, dividing participants into two groups based on their exposure status: non-exposed and occupationally exposed individuals. Categorical variables were expressed as absolute frequencies and percentages, whereas continuous variables were summarized as mean ± standard deviation (SD) or median and interquartile range (IQR: 25th–75th percentile), depending on their distribution. For comparisons between exposure groups, age was assessed using Welch’s *t*-test, categorical variables using Fisher’s exact test, and non-normally distributed continuous variables using the Mann–Whitney U test. Serum levels or activities of oxidative stress biomarkers, including SOD, CAT, GSH, GSSG, and the GSH/GSSG ratio, were compared between exposure groups and between sexes within each exposure group using the Mann–Whitney U test. Distributions were visualized using violin plots with internal boxplots representing the median and interquartile range, and individual points corresponding to each observation.

To characterize multi-element exposure, metal burden indices were constructed from metal concentrations standardized as z-scores. For each individual, the index was calculated as the average of the standardized concentrations, yielding a global metal burden index, an essential metal index, and a toxic metal index. This method enabled a summary of the relative exposure burden and facilitated comparisons among individuals, all while reducing scale differences across metals. Positive index values indicate a metal burden above the sample average, while negative values indicate a burden below the average. Bivariate associations between metals and oxidative stress biomarkers were evaluated using Spearman’s correlation coefficients, which are appropriate for non-normally distributed data and monotonic relationships. Results were presented as heatmaps stratified by exposure group. Metal concentrations were log_10_-transformed. Due to the limited sample size, Principal Component Analysis (PCA) was selected as a parsimonious approach to summarize correlated metal exposures into a small number of interpretable components. PCA was performed to explore co-exposure patterns and reduce the dimensionality of the metal dataset. The first two principal components, PC1_mix and PC2_mix, which together explained more than 50% of the total variability, were used to visualize individual distributions in score plots, and factor loadings were examined to identify the relative contribution of each metal.

To evaluate the joint effect of the metal mixture on oxidative stress biomarkers, Generalized Additive Models (GAMs) were initially used to assess potential non-linear relationships. Because no relevant non-linear effects were observed, multiple linear regression models were then fitted. In these models, oxidative stress biomarkers were considered dependent variables, and the principal components, primarily PC1_mix and PC2_mix, were included as independent variables, with adjustment for age, sex, and exposure status. Regression coefficients (β) and 95% confidence intervals (95% CIs) were estimated. All analyses were conducted in R using version 4.5.1 for Windows (R Foundation for Statistical Computing, Vienna, Austria) using the FactoMineR, factoextra, ggplot2, mgcv, dplyr, openxlsx, and pheatmap packages. Statistical significance was set at *p* < 0.05.

## 3. Results

### 3.1. Sociodemographic Characteristics of Sampled Populations

The study included 74 participants, comprising 49 occupationally exposed and 25 non-exposed individuals. Mean age was comparable between groups, with no significant difference observed. As expected for an artisanal and small-scale gold-mining population, the exposed group was predominantly male, whereas women were more frequently represented in the non-exposed group (14.3% vs. 44.0%, *p* = 0.010). Alcohol consumption was reported by 67.3% of exposed participants and 56.0% of non-exposed individuals, showing no significant difference between groups. Coffee consumption was common in both groups, with 91.8% of exposed participants and 76.0% of controls reporting its use. Median daily coffee intake was similar between groups. Exposed participants reported fewer working hours per week than non-exposed individuals, whereas years in the main occupation were comparable. After collapsing reported occupations into broader categories, exposed participants were distributed across mining extraction and underground operations (32.7%), ore transport and haulage (18.4%), processing and equipment operation (20.4%), and general mining support (28.6%). Because workers in artisanal and small-scale mining often rotate among tasks in response to operational demands, team organization, and production cycles, these occupational categories should be interpreted as predominant roles rather than as mutually exclusive functions. In contrast, non-exposed participants were concentrated in household/domestic work (36.0%), commerce and local services (32.0%), and non-mining technical/manual services (32.0%) (Table 1). Other potential lifestyle-related confounders, including dietary habits, dental fillings, and use of personal protective measures, did not differ significantly between occupationally exposed and non-exposed individuals.

### 3.2. Concentration of Essential and Toxic Elements in Hair Samples

**Figure 2:**
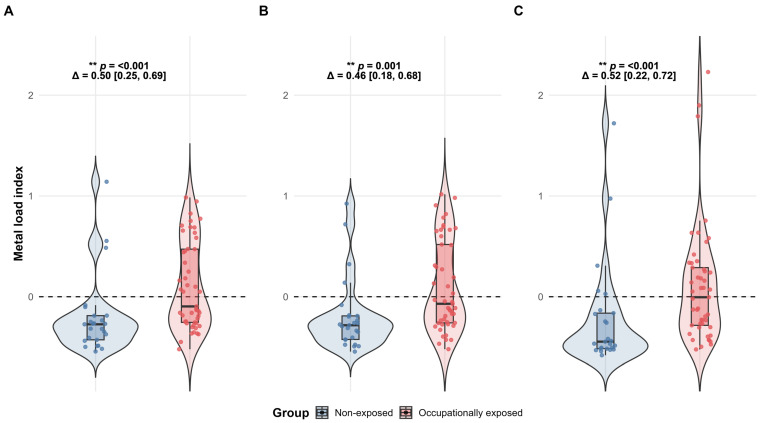
Violin plots show the distribution of metal load indices in non-exposed (blue) and occupationally exposed (red) groups. Panel (**A**) shows the global metal load index, Panel (**B**) the essential elements index, and Panel (**C**) the toxic elements index. The dashed horizontal line marks the zero reference for the standardized index. Violin shapes represent kernel density estimates, boxes show medians and interquartile ranges, and points mark individual observations. Group differences were assessed using the Wilcoxon rank-sum test; effect sizes are reported as Cliff’s delta (Δ) with 95% confidence intervals. Statistical significance is indicated by ** *p* < 0.01.

A multi-element exposure assessment was conducted using ICP-MS analysis on hair samples from occupationally exposed and non-exposed participants. To summarize cumulative internal exposure beyond single-element comparisons, standardized metal load indices were constructed by aggregating z-score-transformed concentrations across predefined element groups. This approach allowed comparison of the overall mixture burden while minimizing the influence of differences in absolute concentration ranges among elements. The global metal load index was significantly higher in the occupationally exposed group, showing a clear rightward shift and broader dispersion relative to non-exposed individuals, with a moderate-to-large effect size (Cliff’s Δ = 0.50, 95% CI: 0.25–0.69; Figure 2A). This pattern indicates a robust population-level separation between exposure strata beyond *p*-values.

When metals were classified based on toxicological classification, distinct and toxicologically relevant patterns emerged. The essential elements index (Figure 2B) exhibited a moderate rightward shift in the occupationally exposed group, suggesting a group-level perturbation of essential metal homeostasis rather than simple nutritional variability. In contrast, the toxic elements index (Figure 2C) showed a marked increase among occupationally exposed individuals. This finding indicates that the observed increase in global metal burden is predominantly reflected in the enrichment of toxic elements rather than isolated changes in essential-element status. Collectively, these findings support the interpretation that occupational exposure is associated with a shift in the overall metal mixture profile, characterized predominantly by enrichment of toxic elements and an altered balance of essential metals.

**Figure 3:**
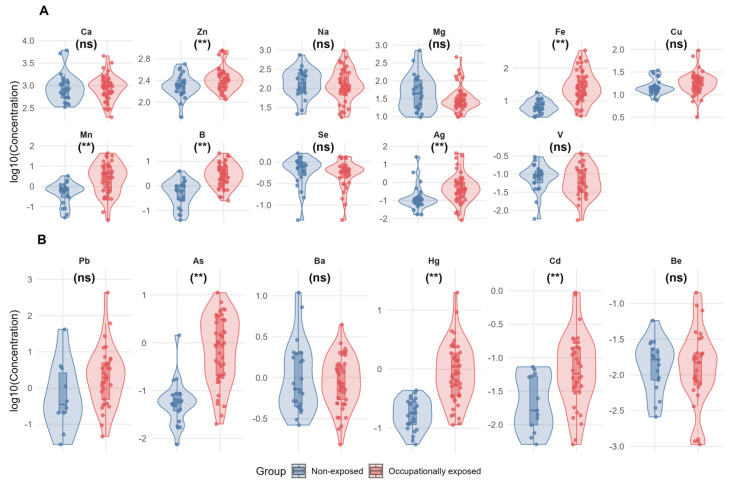
Distribution of log_10_-transformed elemental concentrations in non-exposed (blue) and exposed (red) groups. Panel (**A**) shows essential and nutrient elements (Ca, Zn, Na, Mg, Fe, Cu, Mn, B, Se, Ag, V), while Panel (**B**) shows toxic elements (Pb, As, Ba, Hg, Cd, Be). Violins represent kernel density estimates, boxes indicate medians and interquartile ranges, and points correspond to individual observations. Group differences were assessed using the Wilcoxon rank-sum test; asterisks denote statistical significance (** *p* < 0.01); ns indicates non-significant differences.

To determine which elements most significantly contribute to this pattern, individual metal concentrations were analyzed. Figure 3 displays the distribution of log_10_-transformed elemental concentrations for both occupationally exposed and non-exposed participants. Among essential elements (Figure 3A), Zn, Fe, Mn, B, and Ag showed significantly higher concentrations in occupationally exposed individuals, whereas no significant differences were observed for Ca, Na, Mg, Cu, Se, or V. For toxic elements, As, Hg, and Cd were significantly elevated in the exposed group, whereas Pb, Ba, and Be did not differ between groups (Figure 3B). These elevated concentrations are consistent with ASGM exposure scenarios associated with mining-related activities, and potential uptake via environmental pathways such as dust, water, or food.

When stratified by sex, the distribution of elements showed only limited modification of the overall exposure pattern (Supplementary Table S1). Most elements did not differ significantly between exposed and non-exposed individuals within sex strata, particularly among women. In men, some of the elements identified in the overall analysis—such as Mn, Fe, As, Hg, and Pb—showed higher concentrations in exposed individuals; however, these differences were not consistent across all elements and did not define a distinct pattern separate from the overall multielement profile. For several elements, including Cu, Zn, Ca, Na, Mg, Ba, and Se, no consistent differences by exposure status were observed within either sex. Given the variability in subgroup sizes, particularly among women, these results should be interpreted with caution.

### 3.3. Correlation Structure of the Metal Mixture

**Figure 4:**
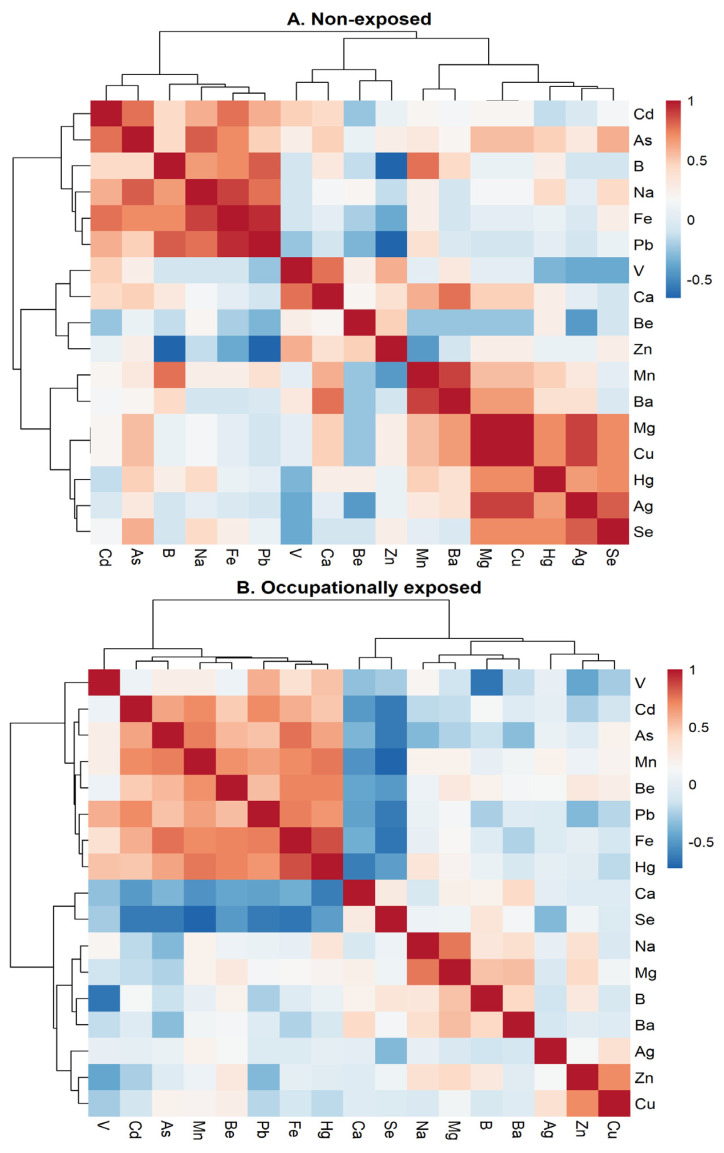
Spearman correlation structure of multielement exposure in non-exposed and occupationally exposed populations. Heatmaps display Spearman correlation matrices with hierarchical clustering performed independently within each group, allowing the identification of group-specific correlation patterns and metal clusters. (**A**) corresponds to non-exposed individuals and (**B**) to occupationally exposed individuals. Red squares indicate positive correlations, while blue squares denote negative correlations.

To explore patterns of co-occurrence among metals, Spearman correlation analysis was conducted. Figure 4 presents a Spearman correlation heatmap with hierarchical clustering, comparing the correlation structures of metals in non-exposed (Figure 4A) and exposed (Figure 4B) individuals. The main purpose of this figure was to determine whether metal exposures occur independently or as part of structured mixtures, thereby providing insights into shared environmental sources and exposure pathways.

The heatmaps revealed distinct differences in the correlation structure of metal concentrations between non-exposed and occupationally exposed groups. Two main patterns emerged from the analysis: (i) The non-exposed population displayed a heterogeneous and fragmented correlation structure, characterized by several small clusters with moderate positive correlations but no dominant multielement pattern. As shown in panel A, metals such as Cd, As, B, Na, Fe, and Pb showed moderate positive associations, while elements including Cu, Hg, Ag, Se, and Mg formed a separate cluster with relatively weaker correlations. Several elements, including Ca, Zn, and Mn, showed limited or inconsistent correlations with the main clusters, suggesting a background exposure scenario in which metals originate from multiple independent sources. (ii) The exposed population showed strong positive correlations among several metals with recognized environmental and toxicological importance (Figure 4B). A prominent cluster composed of Cd, As, Mn, Be, Pb, Fe, and Hg emerged as one of the most notable features of the heatmap. These elements showed consistent positive correlations, suggesting they tend to co-occur in exposed individuals. The simultaneous association of redox-active elements such as Fe and Mn with toxic metals, including As, Pb, Cd, and Hg, highlights a multielemental exposure pattern involving elements commonly reported in mining-affected environments. Taken together, the correlation structure observed in the exposed population suggests a distinct multielemental exposure signature, dominated by associations among Cd, As, Mn, Be, Pb, Fe, and Hg.

This pattern may represent a mixed geogenic and mining-related exposure signature, reflecting the simultaneous occurrence of elements naturally present in mineralized environments and elements mobilized or released during mining activities.

### 3.4. Principal Component Analysis (PCA) of the Element Mixture

**Figure 5:**
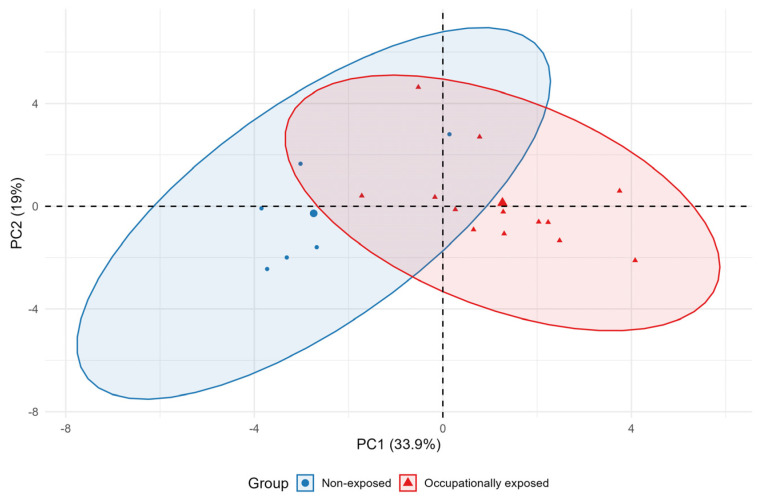
PCA score plot showing the distribution of individuals based on the first two principal components (PC1 and PC2). Ellipses represent the 95% confidence regions for non-exposed and occupationally exposed groups. The dashed vertical and horizontal lines represent the origin of the PCA space (PC1 = 0 and PC2 = 0), dividing the score plot into four quadrants.

The strong correlations observed among several metals suggest the presence of underlying mixture patterns. Therefore, principal component analysis (PCA) was performed to summarize correlated variables and identify the dominant components driving variability in the elemental mixture (Figure 5). This approach also allows the identification of dominant exposure patterns while addressing multicollinearity among elements. The first two principal components explained 52.9% of the total variance in the dataset, with the first principal component (PC1) accounting for 33.9% and the second principal component (PC2) explaining 19.0%.

The projection of individuals onto the space defined by the first two principal components revealed a clear tendency toward separation between the study groups. Non-exposed individuals were mainly located on the negative side of PC1, while exposed individuals tended to cluster on the positive side along the same axis. This pattern shows that the first principal component captures the primary variation in the metal mixture and distinguishes the overall metal profiles between occupationally exposed and non-exposed participants. Although some overlap between groups was observed, the overall distribution of individuals suggests a consistent difference between the multielemental profiles of the two groups.

**Figure 6:**
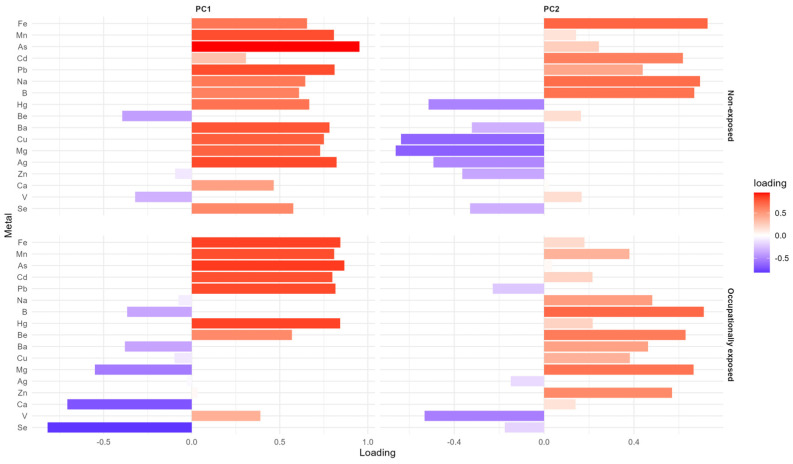
Loadings of the first two principal components (PC1–PC2) obtained from principal component analysis of metal concentrations conducted separately for non-exposed and exposed individuals.

Next, to evaluate whether the multielement mixture showed different structural patterns between groups, PCA was conducted separately for non-exposed and occupationally exposed individuals. The loadings of the first two principal components are shown in Figure 6. PC1 represented the dominant axis of variability in both groups. In non-exposed individuals, PC1 was mainly characterized by positive contributions from As, Mn, Pb, Fe, and Ag, suggesting that these elements defined the main gradient of variation under background exposure conditions. In exposed miners, a similar set of elements contributed to PC1, but the loadings were generally stronger and more evenly distributed, particularly for As, Hg, Pb, Cd, and Fe. Several of these elements, including Hg, Fe, Pb, As, Mn, and Cd, were also part of the main correlated cluster identified in the heatmap analysis. Overall, PC1_mix was interpreted as an integrated multielement exposure pattern reflecting both geogenic background contributions and mining-associated mobilization of toxic elements, rather than as a marker of a single exposure source.

The second principal component (PC2) captured secondary variability within the metal mixture and showed less direct correspondence with exposure-group separation. In non-exposed individuals, PC2 was influenced mainly by Fe, Cd, Na, and B, with negative loadings for Mg, Cu, Ag, and Hg. In exposed miners, PC2 showed stronger positive contributions from B, Be, Mg, Zn, and Cu and a pronounced negative loading for V. Therefore, PC2_mix was interpreted more cautiously as a secondary mixture pattern that may reflect physiological, dietary, nutritional, or background geochemical variability rather than the dominant occupational exposure gradient.

Finally, to formally assess the separation observed in the PCA space, the PC1 scores were compared between exposed and non-exposed individuals using Welch’s *t*-test for independent samples. The analysis revealed a statistically significant difference between groups (t = −5.29, *p* = 0.00026). Non-exposed individuals showed a mean PC1 score of −2.74, whereas exposed individuals exhibited a mean score of 1.26. The 95% confidence interval for the difference between means ranged from −5.67 to −2.34, confirming a significant difference in the dominant metal mixture pattern between the two groups.

### 3.5. Oxidative Stress-Related Biomarkers

**Figure 7:**
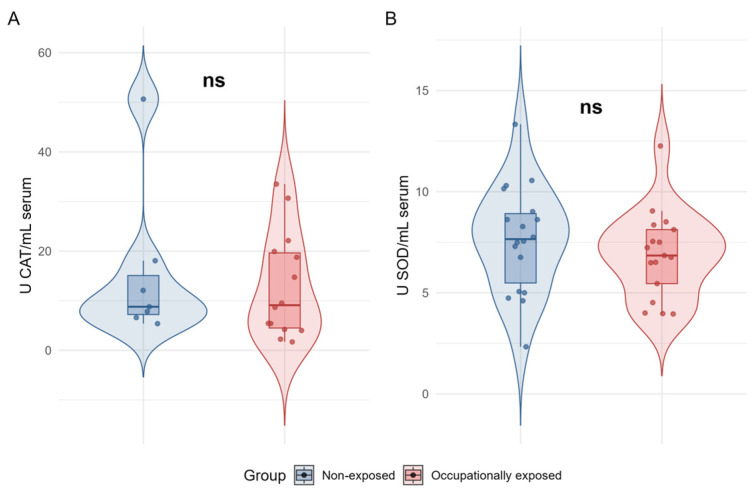
Violin plots of serum antioxidant enzyme activities in non-exposed (blue) and occupationally exposed (red) groups. Panel (**A**) shows CAT levels, and Panel (**B**) shows SOD levels. Boxes indicate medians and interquartile ranges, and points represent individual observations. Group comparisons were performed using the Wilcoxon rank-sum test; “ns” denotes non-significant differences.

After characterizing the cumulative internal burden of elements, we evaluated whether this structured multi-element exposure profile was accompanied by systemic alterations in oxidative stress-related biomarkers. At the overall group level, the major enzymatic antioxidant defenses appeared largely preserved. Activities of catalase (CAT) and superoxide dismutase (SOD) did not differ between exposed and non-exposed individuals, and their distributions showed broad overlap between groups (Figure 7). This pattern was consistent with the adjusted sex-stratified analyses, in which no exposure-related differences were detected for CAT or SOD in either men or women (Supplementary Table S2).

**Figure 8:**
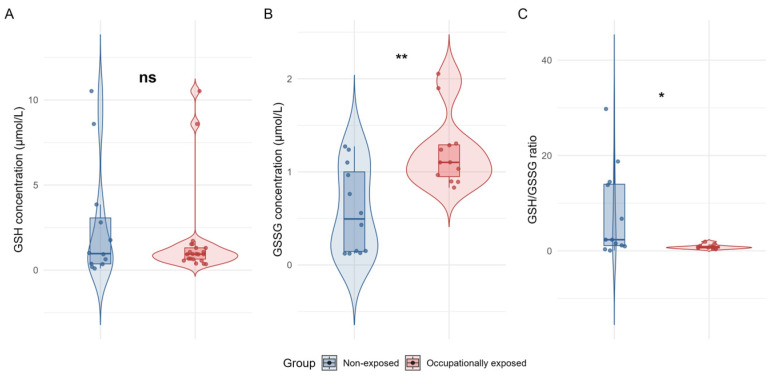
Violin plots of redox biomarkers in non-exposed (blue) and occupationally exposed (red) groups. Panel (**A**) shows reduced glutathione (GSH) concentration, Panel (**B**) shows oxidized glutathione (GSSG) concentration, and Panel (**C**) shows the GSH/GSSG ratio. Violin shapes represent data distributions, boxes indicate medians and interquartile ranges, and points correspond to individual observations. Group differences were evaluated using the Wilcoxon rank-sum test; “ns” denotes non-significant differences, and asterisks indicate statistical significance (* *p* < 0.05; ** *p* < 0.01).

In contrast, glutathione-related parameters showed greater sensitivity to exposure status (Figure 8). Reduced glutathione (GSH) concentrations did not differ significantly between occupationally exposed and non-exposed individuals in the total population or within sex strata, although the distribution showed a visually lower and less dispersed pattern among exposed workers. This non-significant downward shift in GSH was accompanied by significantly higher oxidized glutathione (GSSG) concentrations in exposed individuals, consistent with increased oxidative pressure. Accordingly, the GSH/GSSG ratio was markedly lower in the exposed group, indicating a shift toward a more oxidized glutathione redox status.

Sex-stratified analyses further clarified this pattern. Among men, exposed individuals showed significantly higher GSSG levels compared with non-exposed men, accompanied by a marked reduction in the GSH/GSSG ratio, indicating a shift toward a more oxidized redox state. In contrast, no significant exposure-related differences were observed among women for GSSG or the GSH/GSSG ratio (Supplementary Table S2). Although these results suggest a possible sex-related pattern in glutathione-related responses, they should be interpreted as exploratory, particularly among exposed women, due to the limited sample size in this group.

### 3.6. Association Between Multi-Element Exposure Signature and Oxidative Stress Biomarkers

To explore potential non-linear associations between exposure and oxidative stress biomarkers, generalized additive models (GAMs) were initially fitted. Since no significant non-linear patterns were observed, linear regression models were subsequently used to assess the associations between principal components derived from the multielement exposure profile (PC1_mix and PC2_mix) and oxidative stress biomarkers, adjusting for age and sex (Table 2).

The associations were primarily driven by the main multielement exposure component (PC1_mix), whereas the secondary component (PC2_mix) showed no statistically significant relations with any biomarker. For enzymatic antioxidants, SOD activity decreased along with multielement exposure in both the total sample and occupationally exposed individuals, but no such association was observed in non-exposed individuals. Conversely, CAT activity was not associated with the exposure components, although sex significantly affected CAT levels in the total sample.

In contrast to enzymatic antioxidants, glutathione-related biomarkers exhibited a more differentiated pattern. Total GSH levels showed no significant association with either exposure components or exposure status. However, GSSG levels were elevated among occupationally exposed individuals, suggesting increased oxidative stress in this group. The most consistent associations were observed for the GSH/GSSG ratio, which showed opposite patterns between groups. In the total sample, the ratio increased with PC1_mix, and this positive association was particularly strong among exposed individuals. In contrast, among non-exposed, PC1_mix was negatively associated with the ratio. Age was positively associated with the GSH/GSSG ratio across models, while sex showed a significant association only in the non-exposed groups. Sex-related differences were observed for CAT activity in the total sample and for the GSH/GSSG ratio in the control group. However, these associations were not consistent across biomarkers or exposure groups, suggesting that sex may contribute to baseline biological variability rather than representing a central determinant of the observed exposure-related patterns.

**Figure 9:**
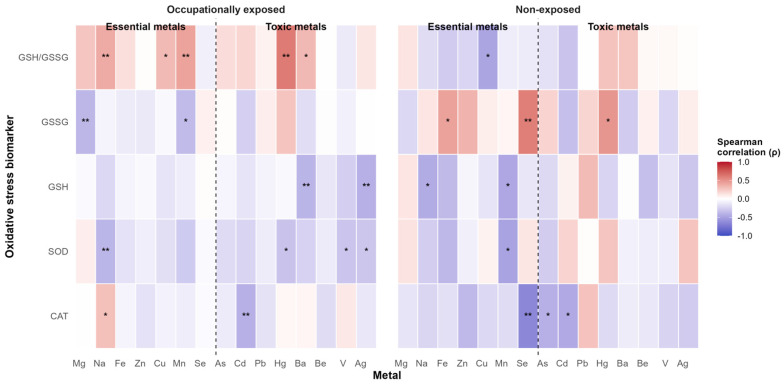
Heatmaps of Spearman correlation coefficients (ρ) between element concentrations and enzymatic and non-enzymatic redox biomarkers in exposed and non-exposed groups. Essential and toxic elements are shown separately within each group. Color intensity indicates the strength and direction of the correlation (red, positive; blue, negative). Asterisks denote statistically significant correlations (* *p* < 0.05; ** *p* < 0.01). Dashed vertical lines separate essential from toxic elements.

Following the regression analyses, which identified PC1_mix and PC2_mix as the main exposure-related components associated with oxidative stress biomarkers, we further explored whether these associations were reflected in individual metal–biomarker relationships. To address this, Spearman correlation analyses were performed between individual metal concentrations and oxidative stress biomarkers, stratified by exposure status (Figure 9).

The results revealed a distinct correlation structure between occupationally exposed miners from the Santurbán páramo and non-exposed individuals from Charta. In occupationally exposed individuals, the dominant pattern was observed for the GSH/GSSG ratio, which showed significant positive correlations with Na (**), Mn (**), Cu (*), Hg (**), and Ba (*). In contrast, GSSG exhibited significant negative correlations with Mg (**) and Mn (*). GSH showed significant negative correlations with Ba (**) and Ag (**). Enzymatic antioxidant activity also showed specific associations: SOD was negatively correlated with Na (**), Hg (*), V (*), and Ag (*), while CAT was positively correlated with Na (*) and negatively correlated with Cd (**).

In non-exposed individuals, the correlation pattern differed in both direction and distribution. The GSH/GSSG ratio showed a significant negative correlation with Cu (*). GSSG exhibited significant positive correlations with Se (**), Fe (*), and Hg (*). GSH showed significant negative correlations with Na (*) and Mn (*), while SOD displayed a significant negative correlation with Mn (*). Finally, CAT showed significant negative correlations with Se (**), As (*), and Cd (*).

Importantly, these individual metal–biomarker associations indicate that the mixture-level effects captured by principal component analysis are only partially recapitulated at the level of single elements. The glutathione-related responses in occupationally exposed individuals were distributed across both principal components, with Mn and Hg aligning with the primary exposure gradient (PC1_mix) and Na, Cu, and Ba contributing through the secondary dimension (PC2_mix). The inverse association between GSSG and Mg further suggests that PC2_mix modulates specific aspects of redox variability. Similarly, the enzymatic responses—particularly the negative correlations of SOD with Hg (a PC1mix element) and with Na and Ag (elements outside the primary cluster)—indicate that SOD inhibition may reflect contributions from multiple exposure dimensions rather than a single dominant metal. 

## 4. Discussion

The findings of this study suggest that the redox response to occupational multielement exposure in the Santurbán páramo should be interpreted in the context of a shared high-altitude hypoxic background. Both occupationally exposed miners and non-exposed individuals were recruited from high-altitude Andean municipalities. The exposed area spanned approximately 2050–3350 m a.s.l., whereas the non-exposed group was recruited from Charta, located at approximately 2474 m a.s.l. This shared high-altitude residential context may influence baseline redox physiology through hypoxia-related metabolic and mitochondrial adaptations [49]. Within this context, the exposed group showed a distinct redox profile rather than a simple amplification of the pattern observed in non-exposed individuals. This suggests that occupational exposure to metal mixtures may impose an additional redox/metabolic burden within the shared high-altitude background, with glutathione-related markers appearing as the most consistently responsive redox compartment in this population.

In this shared high-altitude setting, occupationally exposed miners exhibited a clearly differentiated multielement exposure profile. Compared with non-exposed individuals, exposed workers showed higher concentrations of several elements, particularly Fe, Mn, As, Pb, Cd, and Hg, which were also among the main contributors to the group separation. The metal–metal correlation matrix indicated that these elements did not vary independently but tended to co-vary across individuals, forming a consistent exposure cluster. Principal component analysis supported this structure, as the first principal component of the mixture, PC1_mix, was primarily composed of Fe, Mn, As, Pb, Cd, and Hg and captured the dominant occupational exposure signature. This clustering is geochemically plausible, since Fe and Mn oxides can act as carriers that promote the adsorption, transport, and co-mobilization of trace elements such as As, Pb, and Cd under changing redox conditions [50,51]. The contribution of Hg to this component is also consistent with an anthropogenic input related to gold-extraction activities [52]. Therefore, PC1_mix likely represents an integrated exposure pattern combining geogenic and mining-related sources, rather than the effect of a single isolated element.

The biological implications of this exposure profile are relevant because the elements contributing to PC1_mix may affect redox homeostasis through complementary mechanisms. Fe and Mn are redox-active elements that can participate in reactions leading to reactive oxygen species generation, whereas Cd, Pb, As, and Hg may disrupt antioxidant defenses indirectly through thiol binding, mitochondrial dysfunction, interference with antioxidant enzymes, or displacement of essential cofactors [53,54]. The co-occurrence of these elements within the same exposure component may therefore impose a complex oxidative burden on thiol-dependent and enzyme-dependent antioxidant systems. In contrast, the second mixture component, PC2_mix, captured additional variability involving elements such as Na, Cu, Ba, and Mg, which may reflect dietary, physiological, or secondary geochemical sources. Although this component contributed less clearly to group separation, its associations with glutathione-related markers suggest that secondary exposure or physiological patterns may also modulate redox responses.

The antioxidant enzyme response was not uniform across biomarkers. CAT and SOD activities did not show clear group-level differences, which is consistent with the heterogeneous and context-dependent behavior of enzymatic antioxidant defenses reported in metal-exposed populations [55]. However, the inverse association between PC1_mix and SOD activity among exposed workers suggests that enzymatic antioxidant responses may still be affected along the internal exposure gradient. This finding may reflect partial inhibition of SOD activity under increasing multielement burden, possibly through interactions with sulfhydryl groups or disruption of essential metal cofactors such as Zn, Cu, and Mn [56]. Nevertheless, this interpretation should remain cautious because SOD activity can be influenced by multiple physiological factors, including nutritional status, inflammation, duration of exposure, and adaptive antioxidant regulation.

In contrast to CAT and SOD, glutathione-related parameters showed a more consistent alignment with the exposure pattern. This differential behavior is biologically plausible in a high-altitude environment, where chronic hypoxia may increase the metabolic cost of maintaining redox balance [57]. Hypoxia-related mitochondrial and metabolic adaptations can affect ATP production, cystine availability, and NADPH-dependent glutathione recycling, thereby limiting the conversion of GSSG back to GSH [58,59]. Although these mechanisms were not directly assessed in the present study, they provide a plausible framework for interpreting the GSSG accumulation and the negative associations observed for the GSH/GSSG ratio in the non-exposed high-altitude group. This interpretation is particularly relevant in the Santurbán páramo, where occupational multielement exposure occurs within a high-altitude ecosystem in which oxygen availability may already condition glutathione recycling and antioxidant reserve capacity. Under these conditions, the glutathione system may become more vulnerable to additional toxicant-related demands, particularly when exposure includes metals and metalloids capable of binding thiol groups, impairing mitochondrial function, or interfering with antioxidant enzyme activity.

In the non-exposed group, this background condition may help explain the pattern of GSSG accumulation and the negative associations observed for the GSH/GSSG ratio. However, occupationally exposed miners showed a distinct glutathione-related redox profile. This pattern should be interpreted at two complementary levels: the between-group comparison, which reflects differences in the overall redox state between exposed and non-exposed individuals, and the within-group associations, which describe how glutathione-related markers vary across the internal exposure gradient among exposed workers. This distinction is important because a generalized oxidative shift at the group level may coexist with positive correlations between selected elements and glutathione-related markers within the exposed group, reflecting adaptive or compensatory antioxidant responses in subsets of exposed individuals.

At the between-group level, the lower GSH/GSSG ratio observed in occupationally exposed miners, together with significantly higher GSSG concentrations and a non-significant downward shift in GSH, suggests a shift toward a more oxidized glutathione redox status. This shift may reflect the cumulative oxidative burden imposed by the multielement mixture rather than the effect of any single metal, as the mixture-level exposure captured by PC1_mix encompasses both redox-active (Fe, Mn) and redox-inactive (Cd, Pb, As, Hg) elements whose combined action could exceed the recycling capacity of the glutathione system [20,54,60]. Because GSH can act as both a redox buffer and a metal-binding or metal-conjugating thiol, its availability for antioxidant recycling may be reduced when a substantial fraction is diverted toward metal complexation or detoxification. Thus, the observed glutathione-related changes may reflect a broader thiol-dependent response to multielement exposure, involving both redox regulation and metal-chelating demands. Although Cd, Pb, and As did not show significant individual correlations with glutathione parameters in the exposed group, these redox-inactive metals may still contribute to thiol-dependent redox alteration as part of the aggregate mixture burden, given their capacity to bind sulfhydryl groups and deplete intracellular GSH. Notably, at the individual metal level, Cd, Pb, and As did not show significant correlations with glutathione parameters in the exposed group, suggesting that their contribution to redox disruption may operate through the aggregate burden of the mixture rather than through individually detectable dose–response relationships.

At the within-group level, the positive correlations between the GSH/GSSG ratio and specific elements, including Mn, Hg, Na, Cu, and Ba, may indicate heterogeneity in adaptive redox responses among exposed workers. Individuals with higher levels of certain elements may activate compensatory pathways that support GSH synthesis, GSSG reduction, or broader antioxidant defense mechanisms [60,61,62,63,64]. However, this interpretation should be considered exploratory. Experimental evidence indicates that glutathione responses to toxicants may be biphasic, with moderate exposure levels inducing protective antioxidant responses, whereas higher or sustained exposures may overwhelm these mechanisms and lead to depletion [64,65]. Under this framework, the positive correlations observed within the exposed group may reflect individuals positioned in a compensatory phase of the response rather than evidence of a beneficial effect of metal exposure.

Interindividual variability in this response could also be influenced by genetic or epigenetic differences in glutathione-related pathways, although these mechanisms were not assessed in the present study. Polymorphisms in genes such as GCLC, GCLM, GSTM1, and GSTP1, as well as epigenetic changes affecting glutathione-related gene expression, have been associated with differences in antioxidant capacity and susceptibility to metal-induced oxidative stress [61,65,66]. In addition, several metals, including As, Cd, and Hg, can interact with redox-sensitive signaling pathways such as Keap1/Nrf2, which regulate a broader cytoprotective transcriptional program involving antioxidant defense, detoxification, thiol metabolism, and metal-response pathways, including but not limited to glutathione synthesis and recycling [66]. In a high-altitude context, these pathways may also interact with hypoxia-related signaling, including HIF-1α activation [67,68,69]. However, these mechanisms should be interpreted as biologically plausible hypotheses rather than direct explanations of the present findings.

## 5. Limitations and Future Directions

Several limitations should be considered when interpreting these results: (i) The cross-sectional design does not allow causal inference or determination of the temporal sequence between metal exposure and redox alterations. (ii) The sample size and sex imbalance, particularly the low number of women among occupationally exposed miners, limited statistical power to detect sex-specific effects or exposure-by-sex interactions; therefore, sex-stratified findings should be interpreted as exploratory and may be affected by type II error. (iii) While both groups were permanent residents of high-altitude municipalities and may be considered chronically acclimatized to their residential altitude, ancestry, place of birth, ethnic background, and multigenerational high-altitude residence were not assessed. Thus, participants were not classified as native high-altitude inhabitants or as genetically adapted high-altitude populations. (iv) Both groups lived at high altitude, which strengthens contextual comparability but also means that the non-exposed group should not be interpreted as a low-oxidative-stress reference population. (v) Although information on smoking, alcohol consumption, dietary habits, dental fillings, occupational history, duration of mining-related activities, and use of personal protective measures was collected, lifestyle, nutritional, environmental, and occupational factors may still contribute to interindividual variability in oxidative stress biomarkers and should be further addressed in larger studies. Finally, key molecular and functional components of glutathione regulation, including GCLC/GCLM expression, Nrf2 activation, HIF-1α signaling, GPx, GR, GST activities, metallothionein concentration or gene expression, and NADPH availability, were not measured. Future studies incorporating these markers, together with longitudinal designs and larger sample sizes, would help clarify whether the observed glutathione-related patterns represent early adaptive responses, sustained compensatory mechanisms, metal-chelating responses, or progressive redox impairment in high-altitude ASGM populations. Overall, these findings support the relevance of evaluating occupational exposure in ASGM settings as a multielement mixture rather than focusing exclusively on Hg or on isolated toxic metals. In the Santurbán páramo, the combined presence of Fe, Mn, As, Pb, Cd, and Hg defined a distinctive exposure signature that was accompanied by selective modulation of glutathione-related redox markers. This pattern suggests that redox alterations in high-altitude mining populations may reflect occupational multielement exposure occurring within a hypoxia-related physiological background. From a public health perspective, these results highlight the need for biomonitoring strategies that integrate exposure mixtures, antioxidant biomarkers, and the specific physiological context of high-altitude páramo communities.

## 6. Conclusions

This study shows that gold miners from the Vetas–California district, located near the Santurbán páramo at approximately 2050–3350 m a.s.l., present a distinct multielement exposure profile within a shared high-altitude environmental background. This profile was mainly characterized by Fe, Mn, As, Pb, Cd, and Hg, suggesting the combined contribution of geogenic sources and mining-related mobilization of toxic elements.

Although CAT and SOD activities showed no marked between-group differences, glutathione-related biomarkers were more responsive to exposure status. Higher GSSG concentrations and a lower GSH/GSSG ratio in exposed miners were consistent with a more oxidized glutathione redox status, whereas GSH did not show a statistically significant decrease. This greater sensitivity of the glutathione system may reflect its dual role in redox buffering and thiol-dependent metal binding, as well as its dependence on NADPH-mediated recycling under high-altitude hypoxic conditions.

Overall, these findings support the value of integrated biomonitoring approaches that combine multielement exposure assessment with redox biomarkers in high-altitude ASGM populations. In páramo mining contexts, glutathione-related markers may provide useful early indicators of redox perturbation and should be prioritized in future longitudinal and mechanistic studies.

## Figures and Tables

**Table 1:** Sociodemographic and lifestyle characteristics of the study population according to exposure status.

Variable ^1^	Total	Non-Exposed	Occupationally Exposed	*p*-Value
Full study sample, *n*	74	25	49	—
Age, years	43.54 ± 9.72	43.84 ± 11.26	43.24 ± 8.10	0.830
Female sex, *n* (%)	18 (24.3)	11 (44.0)	7 (14.3)	**0.010**
Male sex, *n* (%)	56 (75.7)	14 (56.0)	42 (85.7)	**0.010**
Alcohol consumption, yes, *n* (%)	47 (63.5)	14 (56.0)	33 (67.3)	0.445
Coffee consumption, yes, *n* (%)	64 (86.5)	19 (76.0)	45 (91.8)	0.078
Cups of coffee/day	2.0 (1.0–3.0)	2.0 (0.1–4.0)	1.0 (1.0–2.0)	0.707
Working hours per week	48.0 (40.0–65.0)	60.0 (48.0–72.0)	40.0 (35.2–48.0)	<0.001
Years in main occupation	13.50 (4.7–25.2)	15.00 (7.0–26.0)	12.00 (3.7–18.0)	0.307
Occupational category				
Mining extraction	16 (21.6)	—	16 (32.7)	
Ore transport/haulage	9 (12.2)	—	9 (18.3)	
Processing/equipment operation	10 (13.5)	—	10 (20.4)	
General mining support	14 (18.9)	—	14 (28.6)	
Household/domestic work	9 (12.2)	9 (36.0)	—	
Commerce and local services	8 (10.8)	8 (32.0)	—	
Non-mining technical/manual services	8 (10.8)	8 (32.0)	—	

^1^ Values are presented as mean ± SD, median (IQR), or *n* (%), as appropriate. Age was compared using Welch’s *t*-test. Female sex, alcohol consumption, and coffee consumption were compared using Fisher’s exact test. Cups of coffee/day, working hours per week, and years in the main occupation were compared using the Mann–Whitney U test. The occupational-category *p*-value reflects the between-group distribution of the grouped occupation variable. Bold indicates a statistically significant difference.

**Table 2:** Linear regression models, including PC1_mix and PC2_mix, for oxidative stress biomarkers.

Biomarker	Group	Variable	β	95% CI	*p*-Value
SOD	Total	PC1_mix	−0.457	−0.877 to −0.036	0.034
		PC2_mix	−0.025	−0.504 to 0.455	0.918
		Exposure	1.790	−0.090 to 3.670	0.062
		Age	0.008	−0.068 to 0.083	0.839
		Sex	0.175	−1.481 to 1.830	0.834
	Non-exposed	PC1_mix	0.005	−0.999 to 1.009	0.992
		PC2_mix	−0.416	−1.054 to 0.222	0.189
		Age	0.148	0.044 to 0.252	0.008
		Sex	0.891	−1.825 to 3.607	0.502
	Occupationally exposed	PC1_mix	−0.487	−0.953 to −0.020	0.041
		PC2_mix	−0.062	−0.741 to 0.617	0.854
		Age	−0.087	−0.190 to 0.016	0.097
		Sex	0.632	−1.438 to 2.702	0.542
CAT	Total	PC1_mix	0.133	−1.741 to 2.007	0.888
		PC2_mix	−1.135	−3.273 to 1.003	0.293
		Exposure	−1.179	−9.564 to 7.206	0.780
		Age	−0.013	−0.348 to 0.323	0.940
		Sex	7.759	0.378 to 15.139	0.040
	Non-exposed	PC1_mix	−0.628	−6.784 to 5.528	0.834
		PC2_mix	−1.810	−5.719 to 2.100	0.346
		Age	−0.111	−0.749 to 0.526	0.720
		Sex	16.080	−0.565 to 32.726	0.058
	Occupationally exposed	PC1_mix	0.188	−1.741 to 2.117	0.845
		PC2_mix	−0.416	−3.221 to 2.390	0.767
		Age	0.159	−0.267 to 0.585	0.456
		Sex	2.678	−5.879 to 11.236	0.532
GSH	Total	PC1_mix	−0.290	−0.792 to 0.212	0.253
		PC2_mix	−0.495	−1.067 to 0.078	0.089
		Exposure	0.862	−1.384 to 3.107	0.446
		Age	−0.031	−0.121 to 0.059	0.496
		Sex	1.608	−0.368 to 3.585	0.109
	Non-exposed	PC1_mix	−0.751	−2.447 to 0.944	0.366
		PC2_mix	−0.597	−1.674 to 0.479	0.261
		Age	0.056	−0.120 to 0.232	0.513
		Sex	1.398	−3.187 to 5.982	0.532
	Occupationally exposed	PC1_mix	−0.189	−0.705 to 0.328	0.466
		PC2_mix	−0.645	−1.397 to 0.107	0.091
		Age	−0.095	−0.209 to 0.019	0.102
		Sex	1.879	−0.414 to 4.172	0.106
GSSG	Total	PC1_mix	−0.027	−0.110 to 0.057	0.528
		PC2_mix	−0.058	−0.154 to 0.037	0.227
		Exposure	0.412	0.037 to 0.786	0.032
		Age	0.002	−0.013 to 0.017	0.754
		Sex	−0.269	−0.599 to 0.060	0.107
	Non-exposed	PC1_mix	0.008	−0.184 to 0.199	0.935
		PC2_mix	−0.084	−0.205 to 0.038	0.167
		Age	0.017	−0.003 to 0.037	0.083
		Sex	−0.398	−0.916 to 0.120	0.124
	Occupationally exposed	PC1_mix	−0.028	−0.127 to 0.072	0.577
		PC2_mix	−0.073	−0.217 to 0.071	0.314
		Age	−0.010	−0.031 to 0.012	0.384
		Sex	−0.125	−0.565 to 0.315	0.570
GSH/GSSG	Total	PC1_mix	1.062	0.087 to 2.038	0.033
		PC2_mix	0.830	−0.282 to 1.943	0.141
		Exposure	−3.514	−7.877 to 0.850	0.113
		Age	0.328	0.154 to 0.503	<0.001
		Sex	0.805	−3.036 to 4.646	0.677
	Non-exposed	PC1_mix	−3.720	−6.494 to −0.946	0.011
		PC2_mix	1.114	−0.647 to 2.876	0.202
		Age	0.353	0.066 to 0.640	0.019
		Sex	−8.476	−15.976 to −0.976	0.029
	Occupationally exposed	PC1_mix	1.712	0.757 to 2.666	<0.001
		PC2_mix	0.702	−0.686 to 2.090	0.3136
		Age	0.286	0.075 to 0.497	0.009
		Sex	3.000	−1.234 to 7.234	0.160

Underlined values indicate statistical significance: *p* < 0.05. Total = regression models fitted to the entire study population, with exposure status included as a covariate. Non-exposed and Occupationally exposed = regression models fitted separately within each exposure subgroup.

## Data Availability

The data presented in this study are available on reasonable request from the corresponding authors. The data are not publicly available due to ethical and privacy restrictions associated with human biomonitoring data. Requests will be evaluated in accordance with institutional ethical guidelines.

## Supplementary Materials

The following supporting information can be downloaded at https://www.mdpi.com/article/10.3390/toxics14060534/s1. Supplementary Table S1. Sex-stratified concentrations of essential and toxic elements in scalp hair samples from occupationally exposed and non-exposed individuals. Supplementary Table S2. Sex-stratified distribution of oxidative stress biomarkers in the serum of occupationally exposed and non-exposed individuals from high-altitude populations. Supplementary Table S3. External standard concentration ranges, method quantification limits, and certified/reference material agreement for trace elements retained in the quantitative hair elemental exposure dataset. Supplementary Table S4. ICP-MS instrumental and analytical conditions for trace-element determination in scalp hair samples.

## Abbreviations

The following abbreviations are used in this manuscript:

ASGM	Artisanal and Small-Scale Gold Mining
ARE	Antioxidant Response Element
CAT	Catalase
NADPH	Nicotinamide Adenine Dinucleotide Phosphate (reduced form)
Nrf2	Nuclear Factor Erythroid 2-Related Factor 2
Fe	Iron
GCLC	Glutamate–Cysteine Ligase Catalytic Subunit
GPx	Glutathione Peroxidase
GR	Glutathione Reductase
GSH	Reduced Glutathione
GSSG	Oxidized Glutathione
GSH/GSSG	Glutathione Redox Ratio
Hg	Mercury
Keap1	Kelch-like ECH-associated protein 1
SOD	Superoxide Dismutase
