# Supplementary material for: Multi-Element Exposure in a High-Altitude Páramo Mining District and Oxidative Stress Biomarkers in Gold Miners
Source: Toxics. 2026 Jun 20;14(6):534. doi: 10.3390/toxics14060534 (PMC13307893; doi:10.3390/toxics14060534)
Supplement: Supplementary file 1 [file toxics-14-00534-s001.zip › toxics-4334774-supplementary.pdf]

Supplementary material

Multi-Element Exposure in a High-Altitude Páramo Mining District and Oxidative Stress Biomarkers in Gold Miners

Lyda Espitia-Pérez [1,*], Luz Helena Sánchez Rodríguez [2], Hugo Brango [3,*], Pedro Espitia-Pérez [1], Dina Ricardo-Caldera [4], Laura Andrea Rodríguez-Villamizar [5] and Álvaro J. Idrovo [5]

[1] Grupo de Investigación Biomédica y Biología Molecular, Facultad de Ciencias de la Salud, Universidad del Sinú, Montería 230001, Colombia; pedrojespitia@unisinu.edu.co (P.E.-P)
[2] Grupo de Investigación en Compuestos Orgánicos de Interés Medicinal, Escuela de Microbiología, Universidad Industrial de Santander, Bucaramanga 680002, Colombia; lsanchez@uis.edu.co (L.H.S.R)
[3] Departamento de Matemáticas, Facultad de Educación y Ciencias, Universidad de Sucre, Sincelejo 700003, Colombia
[4] Grupo de Investigación Enfermedades Tropicales y Resistencia Bacteriana, Facultad de Ciencias de la Salud, Universidad del Sinú, Montería 230001, Colombia; dinaricardoc@unisinu.edu.co (D.R.C.)
[5] Grupo de Investigación en Demografía, Salud Pública y Sistemas de Salud, Departamento de Salud Pública, Escuela de Medicina, Universidad Industrial de Santander, Bucaramanga 680002, Colombia; laurovi@uis.edu.co (L.A.R.-V.); idrovoaj@uis.edu.co (Á.J.I.)
* Correspondence: lydaespitia@unisinu.edu.co (L.E.-P.); hugo.brango@unisucre.edu.co (H.B.)

Contents

Supplementary Table S1. Sex-stratified concentrations of essential and toxic elements in scalp hair samples from occupationally exposed and non-exposed individuals

Supplementary Table S2. Sex-stratified distribution of oxidative stress biomarkers in serum of occupationally exposed and non-exposed individuals from high-altitude populations

Supplementary Table S3. External standard concentration ranges, method quantification limits, and certified/reference material agreement for trace elements retained in the quantitative hair elemental exposure dataset.

Supplementary Table S4. ICP-MS instrumental and analytical conditions for trace-element determination in scalp hair samples.

Supplementary Table S1. Sex-stratified concentrations of essential and toxic elements in scalp hair samples from occupationally exposed and non-exposed individuals

Variable	Sex	Non-exposed		Occupationally exposed		p-value
		Mean ± SD	Median (IQR)	Mean ± SD	Median (IQR)	
Be	Women	0.01 ± 0.01	0.01 (0.01–0.02)	0.01 ± 0	0.01 (0.01–0.01)	0.50
Be	Men	0.02 ± 0.02	0.02 (0.01–0.03)	0.02 ± 0.03	0.02 (0.01–0.02)	0.79
B	Women	0.71 ± 0.55	0.69 (0.27–1.02)	1.2 ± 1	0.7 (0.66–1.52)	0.37
B	Men	1.01 ± 1.17	0.63 (0.23–1.14)	4.93 ± 4.76	3.3 (1.67–6.39)	0.00
Na	Women	189.92 ± 201.26	111.51 (81.11–233.07)	101.17 ± 96.07	91.66 (32.48–116.61)	0.33
Na	Men	177.47 ± 108.2	152.94 (86.4–270.7)	199.09 ± 191.45	132.2 (85.05–268.56)	0.89
Mg	Women	85.85 ± 106.19	47.96 (16.82–107.64)	67.42 ± 76.44	25.68 (21.85–91.14)	0.93
Mg	Men	96.01 ± 181.95	34.86 (18.35–74.26)	44.58 ± 71.57	27.56 (20.21–40.83)	0.41
Ca	Women	1275.98 ± 1365.43	851.42 (671.61–1110.77)	1048.45 ± 631.49	974.13 (623.66–1593.24)	1.00
Ca	Men	1170.7 ± 1460.48	718.71 (533.05–1272.93)	1069.04 ± 791.98	938.26 (631.38–1197.63)	0.52
V	Women	0.1 ± 0.04	0.09 (0.07–0.11)	0.03 ± 0.02	0.03 (0.02–0.03)	0.00
V	Men	0.11 ± 0.09	0.09 (0.05–0.14)	0.11 ± 0.1	0.08 (0.04–0.15)	0.96
Mn	Women	0.89 ± 1.03	0.59 (0.18–1.2)	1.44 ± 1.8	0.48 (0.25–1.79)	0.74
Mn	Men	0.7 ± 0.38	0.75 (0.55–0.88)	8.29 ± 10.94	4.19 (0.89–10.29)	0.00
Fe	Women	6.95 ± 4.09	6.34 (4.59–7.58)	8.12 ± 4.6	5.36 (5.02–10.57)	0.72
Fe	Men	7.42 ± 3.11	5.91 (5.12–10.71)	58.09 ± 69.3	30.17 (16.23–71.62)	0.00
Cu	Women	16.18 ± 7.43	13.33 (12.26–14.67)	12.48 ± 5.67	11.55 (8.2–15.21)	0.15
Cu	Men	16.16 ± 7.73	13.9 (11.27–17.99)	22.13 ± 15.59	19.08 (14.55–24.35)	0.06
Zn	Women	206.34 ± 75.61	191.15 (169.04–214.28)	432.71 ± 278.6	363.9 (247.25–586.5)	0.10
Zn	Men	237.92 ± 129.15	211.9 (149.98–310.91)	288.83 ± 175.69	250.69 (188.91–311.76)	0.31
As	Women	0.18 ± 0.44	0.05 (0.03–0.06)	0.1 ± 0.07	0.07 (0.05–0.15)	0.49
As	Men	0.07 ± 0.05	0.06 (0.05–0.09)	2.06 ± 2.28	1.19 (0.36–3.29)	0.00

Hg	Women	0.24 ± 0.12	0.24 (0.16–0.31)	0.8 ± 1.49	0.17 (0.15–0.42)	0.79
Hg	Men	0.16 ± 0.09	0.13 (0.1–0.18)	1.87 ± 3.25	1.02 (0.54–1.9)	<u>0.00</u>
Se	Women	0.72 ± 0.32	0.77 (0.54–0.99)	0.7 ± 0.29	0.73 (0.55–0.77)	0.81
Se	Men	0.77 ± 0.44	0.78 (0.48–0.96)	0.62 ± 0.35	0.61 (0.4–0.76)	0.22
Cd	Women	0.02 ± 0.02	0.01 (0.01–0.02)	0.01 ± 0.01	0.01 (0.01–0.01)	0.34
Cd	Men	0.04 ± 0.03	0.05 (0.02–0.06)	0.14 ± 0.22	0.07 (0.04–0.15)	0.13
Ba	Women	1.66 ± 1.98	0.97 (0.45–1.99)	1.03 ± 0.59	0.91 (0.7–1.33)	0.72
Ba	Men	1.7 ± 2.77	0.65 (0.46–1.71)	1.12 ± 0.82	0.82 (0.5–1.42)	0.66
Pb	Women	11.53 ± 20.23	2.16 (0.23–13.46)	0.25 ± 0.18	0.34 (0.19–0.35)	0.63
Pb	Men	0.91 ± 1.31	0.32 (0.21–0.95)	20.3 ± 78.76	2.39 (1–5.81)	<u>0.02</u>
Ag	Women	2.48 ± 7.82	0.11 (0.04–0.16)	0.55 ± 0.53	0.4 (0.09–0.95)	0.25
Ag	Men	0.46 ± 0.99	0.1 (0.07–0.15)	3.18 ± 8.46	0.38 (0.18–1.05)	<u>0.01</u>

Values are presented as mean ± standard deviation (SD) and as median (interquartile range, IQR) and correspond to elemental concentrations expressed as μg/g hair. Hair samples were subjected to nitric-acid extraction and diluted to a final volume of 10 mL before ICP-MS analysis; the nominal preparation factor was 10 mL final extract per approximately 200 mg of hair. Comparisons between occupationally exposed and non-exposed individuals within each sex stratum were performed using the Mann–Whitney U test. All tests were two-tailed, and p-values < 0.05 were considered statistically significant (underlined).

Supplementary Table S2. Sex-stratified distribution of oxidative stress biomarkers in serum of occupationally exposed and non-exposed individuals from high-altitude populations

Biomarker	Sex	Non Exposed Mean ± SD	Non Exposed Median (IQR)	Exposed Mean ± SD	Exposed Median (IQR)	p-value
SOD (U/mg protein)	Women	7.47 ± 1.82	7.65 (6.74–8.62)	8.02 ± 0.56	8.02 (7.54–8.51)	0.95
SOD (U/mg protein)	Men	7.77 ± 3.25	7.88 (5.44–9.98)	6.73 ± 2.25	6.76 (4.98–7.81)	0.31
SOD (U/mg protein)	Total	7.64 ± 2.64	7.65 (5.48–8.92)	7.01 ± 2.07	7.23 (5.97–8.24)	0.27
CAT (U/mg protein)	Women	23.11 ± 24.01	12.08 (9.34–31.37)	15.87 ± 12.56	12.27 (7.86–20.28)	0.85
CAT (U/mg protein)	Men	10.01 ± 5.56	8.3 (7.21–11.1)	12.39 ± 9.5	9.51 (4.82–19.35)	0.85
CAT (U/mg protein)	Total	15.63 ± 16.02	8.78 (7.21–15.08)	13.32 ± 10.04	9.51 (5.42–19.35)	0.94
GSH (µmol/L)	Women	3.84 ± 3.54	3.33 (2.13–5.04)	1 ± 0.06	1 (0.98–1.02)	0.30
GSH (µmol/L)	Men	1.97 ± 3.49	0.79 (0.37–1.2)	1.78 ± 2.71	0.94 (0.62–1.36)	0.47
GSH (µmol/L)	Total	2.6 ± 3.47	0.97 (0.37–3.07)	1.65 ± 2.48	0.95 (0.67–1.31)	0.98
GSSG (µmol/L)	Women	0.7 ± 0.64	0.69 (0.14–1.25)	1 ± 0.03	1 (0.99–1.01)	0.98
GSSG (µmol/L)	Men	0.53 ± 0.39	0.49 (0.14–0.81)	1.26 ± 0.41	1.17 (0.95–1.3)	<u>0.00</u>
GSSG (µmol/L)	Total	0.58 ± 0.47	0.49 (0.14–1)	1.19 ± 0.37	1.07 (0.97–1.27)	<u>0.00</u>
GSH/GSSG ratio	Women	13.83 ± 13.13	12.75 (5.08–21.5)	1 ± 0.03	1 (0.99–1.01)	0.30
GSH/GSSG ratio	Men	4.62 ± 5.91	1.96 (1.1–5.22)	0.82 ± 0.49	0.72 (0.51–0.76)	<u>0.02</u>
GSH/GSSG ratio	Total	7.69 ± 9.48	2.35 (1.1–13.98)	0.87 ± 0.42	0.76 (0.63–1)	<u>0.01</u>

Values are presented as mean ± standard deviation (SD) and median (interquartile range. IQR). Comparisons between occupationally exposed and non-exposed individuals were performed using the Mann–Whitney U test. p-values < 0.05 were considered statistically significant (underlined).

Abbreviations: SOD. superoxide dismutase; CAT. catalase; GSH. reduced glutathione; GSSG. oxidized glutathione; GSH/GSSG. glutathione redox ratio.

Supplementary Table S3. External standard concentration ranges, method quantification limits, and certified/reference material agreement for trace elements retained in the quantitative hair elemental exposure dataset.

Element	External calibration/checking range in measured extract (μg/L)	Method LOQ/LQ (μg/g hair)	Certified/reference hair material	Experimental value, mean ± SD (μg/g hair)	Certified/reference interval (μg/g hair)
Ca	0–1,000,000	29	IAEA-086	1127 ± 35	1010–1230
Zn	0–10,000	0.2	NIES CRM No. 13	170 ± 16	146–197
Na	0–1,000,000	11	NCS DC 73347	78.23 ± 5.82	61–117
Mg	0–1,000,000	0.5	IAEA-086	162.42 ± 10	150–200
Fe	0–1,000,000	0.5	NCS DC 73347	28.9 ± 2.1	24–48
Cu	0–10,000	0.04	ERM DB001	28.9 ± 2.2	24–42
Mn	0–10,000	0.02	IAEA-086	9.98 ± 0.3	8.8–10.4
B	0–92	0.030	NCS DC 73347	2.39 ± 0.15	1.75–4.05
Se	0–10,000	0.01	NIES CRM No. 13	2.08 ± 0.2	1.4–2.2
Ag	0–10,000	0.01	NIES CRM No. 13	0.12 ± 0.03	
V	0–10,000	0.01	NCS DC 73347	0.32 ± 0.02	0.09–0.91
Pb	0–10,001	0.01	ERM DB001	1.98 ± 0.02	1.7–2.6
As	0–52	0.01	ERM DB001	0.038 ± 0.02	0.03–0.06
Ba	0–10,000	0.02	NCS DC 73347	12.03 ± 0.33	10–12.8
Hg	0–3,333	0.01	NCS DC 73347	0.463 ± 0.006	0.44–0.90
Cd	0–10,000	0.01	NIES CRM No. 13	0.24 ± 0.05	0.16–0.30
Be	0–10,000	0.001	NCS DC 73347	0.1045 ± 0.003	0.094–0.126

External standard concentration ranges correspond to the standard dilutions measured for ICP-MS calibration/checking and are expressed as μg/L in the measured extract. Method quantification limits (LOQ/LQ), experimental CRM/reference material values, and certified/reference intervals are expressed in the same units as the final hair concentrations, μg/g hair. Agreement was defined as the experimental mean falling within the certified/reference interval reported for each material. Only elements retained in the quantitative exposure analyses of the present manuscript are shown. For Ag, the experimental value is reported because no certified/reference interval was available in the analytical record. LOQ/LQ, method quantification limit; CRM, certified reference material.

Supplementary Table S4. ICP-MS instrumental and analytical conditions for trace-element determination in scalp hair samples.

Panel A. General instrumental and analytical conditions

Parameter	Reported condition
Biological matrix	Scalp hair
Analytical matrix	Nitric-acid hair extract
Instrument	Agilent 7500-A ICP-MS
Software	ChemStation G1834B, version B.03.02
Sample introduction	Peristaltic pump
Nebulizer	Concentric nebulizer
Internal standards	Scandium and yttrium
Final internal standard concentration	90.9 µg/L
Acquisition/reading scheme	Initial stabilization followed by duplicate analytical readings
Calibration	External calibration using certified multielement standards and mercury-specific ICP-MS standard
QA/QC	Procedural blanks, calibration verification solutions, duplicate measurements, and certified/reference human hair materials

Panel B. Monitored m/z values and internal standard correction.

Element	Monitored m/z	Internal standard correction
Be	9	Sc/Y
B	11	Sc/Y
Na	23	Sc/Y
Mg	24	Sc/Y
Ca	43	Sc/Y
V	51	Sc/Y
Mn	55	Sc/Y
Fe	56	Sc/Y
Cu	63	Sc/Y
Zn	66	Sc/Y
As	75	Sc/Y
Se	82	Sc/Y
Ag	107	Sc/Y
Cd	111	Sc/Y
Ba	137	Sc/Y
Hg	202	Sc/Y
Pb	208	Sc/Y

Only elements retained in the quantitative exposure analyses of the present manuscript are shown. Sc, scandium; Y, yttrium; CRM, certified reference material; QA/QC, quality assurance/quality control.
